# A Non-Invasive Technique for Long-Term Monitoring of Gastroesophageal Reflux—A Pilot Study

**DOI:** 10.3390/s23239459

**Published:** 2023-11-28

**Authors:** Marco Laracca, Gianfranco Miele, Luca Podestà, Silvia Sangiovanni

**Affiliations:** 1 Department of Astronautics, Electrical and Energetics Engineering (DIAEE), Sapienza University of Rome, Via Eudossiana 18, 00184 Rome, Italy; marco.laracca@uniroma1.it (M.L.); luca.podesta@uniroma1.it (L.P.); 2 Department of Electrical and Information Engineering, University of Cassino and Southern Lazio, 03043 Cassino, Italy; g.miele@unicas.it

**Keywords:** gastroesophageal reflux, bio-impedance measurement, biomedical measurement

## Abstract

Many people suffer from gastric or gastroesophageal reflux disorder (GERD) due to a malfunction of the cardia, the valve between the esophagus and the stomach. GERD is a syndrome caused by the ascent of gastric juices and bile from the stomach. This article proposes a non-invasive impedance measurement method and demonstrates the correlation between GERD and impedance variation between appropriately chosen points on the patient’s chest. This method is presented as an alternative to the most widely accepted diagnostic techniques for reflux, such as pH-metry, pH-impedance measurement, and esophageal manometry, which are invasive because they use a probe that is inserted through a nostril and reaches down to the esophagus.

## 1. Introduction

Gastroesophageal reflux disorder (GERD) is a syndrome caused by the movement of the stomach’s contents into the esophagus or mouth, causing discomfort or complications [1]. This is due to an incontinence of the cardia, a valve located at the point of separation between the esophagus and the stomach. This valve typically prevents the reflux of gastric contents in the opposite direction with respect to the typical path of food ingestion. If it is damaged, it loses its function and can cause malfunctions of the digestive system and serious disorders such as esophagitis (i.e., inflammation of the esophagus), stricture (narrowing of the diameter of the esophagus), and Barrett’s esophagus, in which some parts of the esophageal mucosa undergo a transformation (metaplasia) [1,2].

GERD is a fairly common problem in both sexes. Many studies have evidenced that its prevalence has increased during the past two decades, although it is difficult to quantitatively estimate the number of cases of GERD because people seeking health care for this pathology probably represent only a very small fraction of people affected by GERD [3,4].

Typically, GERD is diagnosed clinically, which is a cost-effective solution, rather than proceeding directly to endoscopic or alternative diagnostic testing [5,6,7,8,9,10,11,12,13,14,15].

Endoscopic examination (gastroscopy) is an invasive technique. It is the visual inspection of the esophageal canal as far as the stomach using a flexible endoscope. This exam is essential to ascertain the existence and severity of esophagitis and its complications, but it does not allow the patient to be monitored over time to evaluate when and how the reflux occurs. Continuous monitoring of the patient can be made using other methods such as pH-metry, pH-impedance measurement, esophageal manometries, Bravo PH monitoring, and Multichannel Intraluminal Impedance (MII) [5,6,7,8,9,10,11,12]. Unfortunately, all these techniques are invasive since they require a wireless pH capsule or the presence of a catheter that is inserted via the nose and reaches the esophagus. Each one of these techniques is characterized by a different methodology, allowing for different monitoring and diagnosis capabilities. These techniques need to be performed in an ambulatory environment, and there is still no solution that allows for the detection of all types of refluxes.

Further improvement in the technologies used for GERD diagnosis is necessary because it could allow for early diagnosis, potentially decreasing the incidence of more serious complications associated with GERD. In addition to accuracy in its diagnostic capability, the simplicity of the test’s execution and its invasiveness are key features to be considered in the development of novel GERD diagnosis techniques, especially for pediatric patients. Among the non-invasive monitoring techniques, in [13,14,15], novel methods based on salivary pepsin-level measurement were proposed. Unfortunately, these solutions showed a diagnosis rate of less than 50%, significantly limiting their applicability.

In this context, we propose a new non-invasive technique for GERD diagnosis. It is based on impedance measurement of the patient’s chest using standard electrodes attached to suitable points. All the measurement devices are located outside the patient’s body, granting non-invasiveness. The proposed method can be engineered into a portable solution, allowing the patient to be monitored over a long period, during daily activities.

After a brief review of the current methods and novel technologies for GERD diagnosis in Section 2, the proposed system is described in Section 3. The measurement procedure and the obtained measurement results are reported in Section 4 and Section 5, respectively. Finally, the conclusions and future developments are reported in Section 6.

## 2. GERD Monitoring Methods and Technologies

Long-term patient monitoring is an important task for a detailed GERD diagnosis. It allows us to evaluate when and how the reflux occurs, the number of episodes registered over time (typically 24–48 h), and the time relationship between reflux episodes and symptoms.

**Figure 1:**
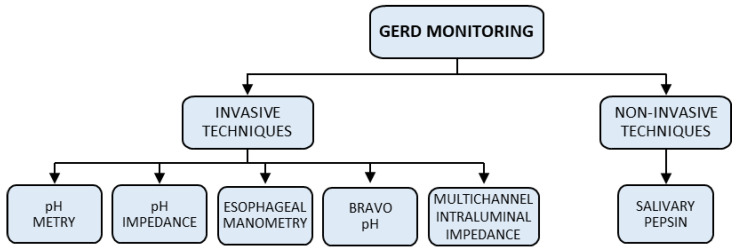
An overview of methods for continuous GERD monitoring.

**Figure 2:**
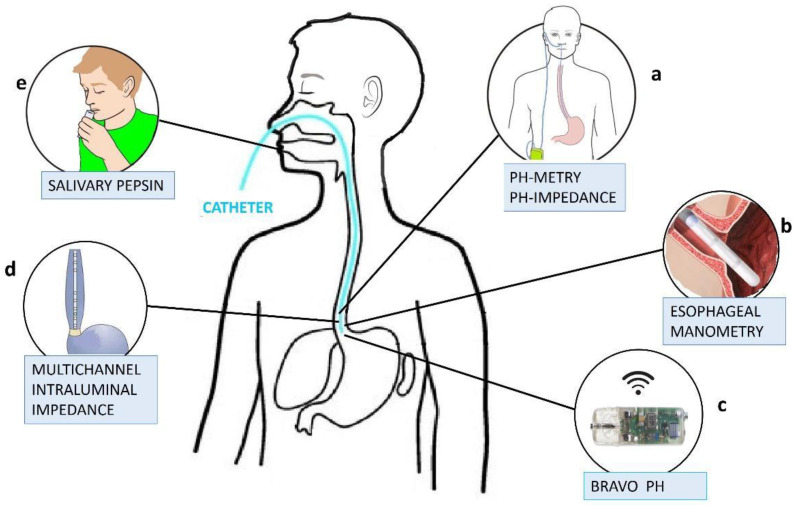
Schematic of the working principle of the invasive and non-invasive GERD monitoring techniques. The light blue device depicts a catheter inserted via the patient’s nostril, reaching down to the esophagus (different catheter technologies are used to apply the different invasive GERD monitoring methods).

Methods for the continuous monitoring of a patient can be divided into two main classes: invasive and non-invasive methods (see Figure 1). While different invasive techniques such as pH-metry, pH-impedance measurement, esophageal manometries, Bravo PH monitoring, and Multichannel Intraluminal Impedance (MII) [5,6,7,8,9,10,11,12] are commonly used in the medical environment, only one non-invasive technique, based on the measurement of the salivary pepsin level, has been proposed in the literature [13,14,15]. Figure 2 shows an overview of both invasive and non-invasive techniques. Details of these techniques are reported in the next two subsections.

### 2.1. Invasive Techniques

pH-metry is a method based on the measurement of the potential of hydrogen (pH) in the esophagus. pH values are generally used to specify the acidity or basicity of an aqueous solution; thus, they can be used to estimate the acidity level in the esophagus over the course of a full day. Typically, when a reflux event happens, the amount of acid in the esophagus increases, with a consequent decrease in the measured pH. This method is based on the insertion of a small probe via a nostril, reaching the esophagus. The probe is then connected to a small and light device, tied to a belt or worn on the shoulder, that records the pH values (Figure 2a). During the monitoring process, the patient should try to perform their usual daily activities, including eating meals as normal. In summary, the pH-metry technique is an invasive solution to monitor reflux events due to the need for a probe inserted via the nostril to the esophagus. This causes irritation in the throat, and it is not able to detect non-acid reflux events.

pH-impedance measurement is a more recent development for the completion and improvement in the information provided by pH-metry. It is now considered the most reliable method to identify gastroesophageal reflux because it allows us to recognize any episode of reflux and to define its composition (acidic, basic, or neutral).

The method is based on the same architecture as pH-metry, and it is composed of a probe, inserted via the nostril and reaching the esophagus, connected to a portable device that contemporarily monitors both the impedance and the pH (Figure 2a).

Esophageal manometry is an examination type that is also carried out with the use of a trans-nasal catheter (Figure 2b) equipped with various sensors that allow for the study of functional alterations in the motility of the entire upper digestive tract, forming a basis for the prevention and diagnosis of gastroesophageal reflux.

A new technique based on the Bravo pH monitoring system was recently developed. This technique consists of placing in the esophagus a pH-sensing wireless capsule, about the size of a gel cap (Figure 2c), by means of esophagogastroduodenoscopy. The capsule senses the pH in the esophagus and sends, wirelessly, the measured data to a wearable recording device. The capsule is then automatically excreted in the feces within the next 7–10 days, on average. This technique is characterized by better comfort, due to the lack of a catheter inserted via the nostril for the whole monitoring time, but it is still an invasive technique: the patient’s throat might be a little sore, and they might feel like something is in their throat.

There are other possible risks. These include damage to the tissue of the esophagus or intestines, and possible bleeding. Finally, this technique is characterized by the same limitations as pH-metry since it is not able to detect non-acid reflux events.

Multichannel Intraluminal Impedance (MII) is a catheter-based method to evaluate esophageal function and gastroesophageal reflux (Figure 2c). The intraluminal bolus movement within the esophagus is detected by means of the impedance change produced by the presence of bolus inside the esophageal lumen and measured via a pair of metallic rings mounted on the catheter.

Impedance measurement at multiple sites (multichannel) permits an evaluation of the direction of bolus movement by means of an analysis of the time differences in bolus entry and exit.

MII is usually performed in combination with manometry (to provide information on the functional component of manometrically detected contractions) or pH testing (to allow for the detection of gastroesophageal reflux at all pH levels, both acid and non-acid).

The last analyzed invasive technique is Mucosal Impedance Testing (MIT), which can be considered an evolution of the MII method. It is not a long-term monitoring technique, allowing us to assess mucosal changes and to diagnose GERD in real time without the need for prolonged ambulatory monitoring [9]. The test is performed by using a catheter to measure the conductivity of the esophageal epithelium directly, in a matter of seconds, during endoscopy, preventing the need for prolonged and cumbersome ambulatory pH or impedance monitoring. MIT values have been shown to better differentiate GERD from eosinophilic esophagitis and normal patients with improved operative characteristics as compared to pH monitoring [10,11]. Outcome studies are still needed to determine whether MIT can predict the response to Proton Pump Inhibitors (PPI) or surgery, which currently limits its ability to become the definitive tool for the diagnosis of GERD [12].

### 2.2. Non-Invasive Techniques

Salivary pepsin-level measurement has been proposed as a non-invasive technique to diagnose GERD [13,14,15]. Pepsin is secreted by the chief cells and activated in acidic gastric secretions; it is then deactivated at pH 7.0 and reactivated after re-acidification. For this reason, pepsin is one of the primary constituents of reflux fluid and can enter the oral cavity when reflux occurs, mixing with saliva.

By measuring the change in the pepsin concentration of saliva, GERD can then be predicted, giving salivary pepsin-level measurement techniques a promising role in the diagnosis of GERD.

Thanks to the non-invasive nature of this test, it would be ideal for the diagnosis of pediatric GERD or extraesophageal reflux. Unfortunately, current data are inconclusive or negative in these cases [14,15]. The limitations of this technique can be summarized as a lack of standardization of the sampling protocols and differences in reference normal values from manufacturers, leading to difficulties in accurate data collection and analysis. Moreover, only 45–50% of patients with known GERD have positive salivary pepsin results.

### 2.3. Final Considerations

In conclusion, invasive monitoring techniques create discomfort and result in reduced oral intake due to trans-nasally positioned catheters, reducing sensitivity and patient compliance.

The only proposed non-invasive technique is still unreliable; thus, there is a need for a simple and efficient method to diagnose GERD without the use of uncomfortable catheter-based systems.

## 3. The Proposed Non-Invasive Measurement Method

Impedance measurements are typically used for medical diagnostics in several applications. These techniques exploit the correlation between physio-pathological events and impedance variations between two suitably chosen points of the patient under examination. Among all the possible configurations of this methodology, some of them can be considered non-invasive as a result of the use of electrodes attached to the patient’s skin [16,17,18,19,20,21].

**Figure 3:**
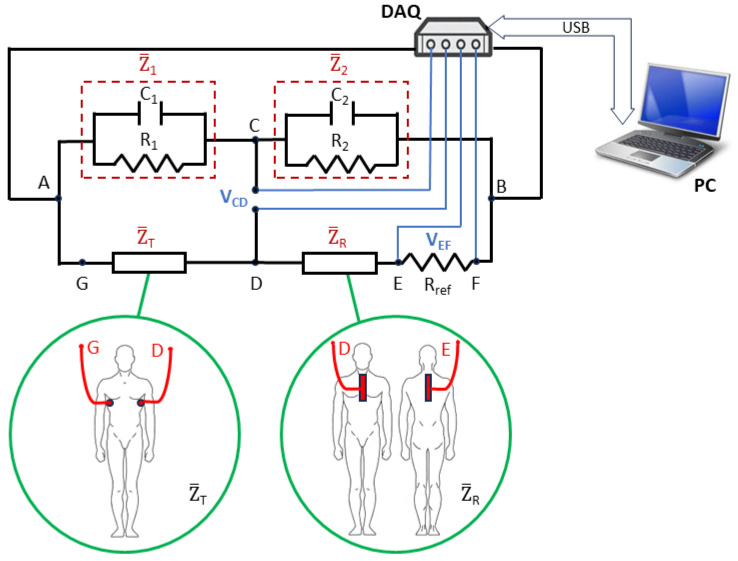
The proposed measurement solution.

The proposed idea for the non-invasive detection of gastric reflux is based on the measurement of the impedance of the gastroesophageal tract using two pairs of electrodes attached to the patient’s skin on the body exterior, suitably positioned as shown in Figure 3. The first two electrodes are positioned in the anterior and posterior areas of the trunk from the base of the neck to the stomach. These allow for the measurement of the impedance of the esophageal tract, named ${\underline{Z}}_{R}$ (hereafter, any generic complex quantity is identified with an overbar notation). The other two electrodes are positioned at the intersection of the medial axillary line with the fifth intercostal space (measuring the impedance there, named ${\underline{Z}}_{T}$). These are used to compensate for the artifacts related to breathing, diaphragmatic contractions, or any other movements of the patient. The chosen electrodes are flexible pre-gelled self-adhesive ones with a rectangular shape. Regarding the dimensions of the electrode pairs, 8 cm × 20 cm and 3 cm × 7 cm were chosen for the ${\underline{Z}}_{R}$ and ${\underline{Z}}_{T}$ measurements, respectively. The electrode pair related to the gastroesophageal tract is larger than the other to cover the overall area under investigation.

To compensate for artifacts due to breathing, diaphragmatic contractions, and body movements during impedance measurement of the esophageal tract, a suitable AC bridge configuration is used for the impedance measurement. In addition, the proposed solution allows for good sensitivity, offering an accurate measurement and reliable results.

Both impedances ${\underline{Z}}_{T}$ and ${\underline{Z}}_{R}$ are formed by the parallel of a resistance and a capacitance. The resistance R_rif_ is the reference resistance used for the measurement of the current flowing in the branch containing ${\underline{Z}}_{R}$.

Impedances ${\underline{Z}}_{1}$ and ${\underline{Z}}_{2}$ are composed of a variable resistance in parallel to a variable capacitance (R_1_//C_1_ and R_2_//C_2_, respectively), and they are used to balance the bridge when it is not stressed by a reflux episode. The orders of magnitude of ${\underline{Z}}_{1}$ and ${\underline{Z}}_{2}$ are established to have good sensitivity to the bridge; therefore, ${\underline{Z}}_{1}$ and ${\underline{Z}}_{2}$ are of the same order of magnitude as ${\underline{Z}}_{T}$ and ${\underline{Z}}_{R}$ in the absence of both reflux and artifacts. These values, as reported in the literature [19], range from 500 Ω to 1300 Ω and from −60° to −40° for the impedance module and phase, respectively.

The values of ${\underline{Z}}_{T}$ and ${\underline{Z}}_{R}$ depend on the different chest characteristics of the patient or on any other factor that can influence the impedance of the chest (such as the presence of esophagitis). It is worth noting that since the influence of these factors on the impedance is a systematic effect, it will be compensated by the bridge balance phase.

The adjustment of these resistances and capacities is only carried out once when the measurement system is installed on the patient.

As shown in Figure 3, the proposed measurement system is completed with a data acquisition (DAQ) board (National Instruments NI USB-6251) that allows for both the generation of the supply signal of the bridge (*V_AB_*) and the acquisition of the voltage signals (*V_CD_* and *V_EF_*) using a sampling frequency of 200 kHz at 16 bits. The DAQ board is connected and powered via a USB interface to a battery-powered notebook (PC), allowing for the safe use of the developed measurement system on the human body.

As far as the choice of frequency to be used for the stimulus signal, different constraints must be taken into account.

The first is related to the typical resistive/capacitive behavior of human tissues. As the frequency increases, the resistive behavior decreases and the capacitive behavior increases, and vice versa. Thus, the frequency choice of the stimulus signal to be used affects the tissue’s resistive/capacitive behavior, allowing it to have a resistive or a capacitive prevalence that can affect the reflux detection capability.

The second is related to the signal-to-noise ratio of the detected signals, which increases with the amplitude of the stimulus current. It is well known that the maximum current amplitude to be used increases with the frequency, as also defined by the international safety standard concerning general requirements for medical electrical equipment [22].

Considering these two constraints, a signal frequency of 10 kHz was chosen to power the bridge. Consequently, to comply with the standard [22], the maximum allowed injectable current value is 1 mA rms.

To satisfy this limit, based on the peak-to-peak bridge power supply value of 4 V and based on the minimum values that can be assumed by ${\underline{Z}}_{T}$ and ${\underline{Z}}_{R}$, R_ref_ equal to 350 Ω was suitably chosen.

The notebook (PC) manages the overall measurement station by means of suitable software written in the LabVIEW^TM^ (2015) environment.

The developed software also allows for the processing the acquired signals, evaluating the current ${\underline{I}}_{ref}$ circulating in both $R_{ref}$ and ${\underline{Z}}_{R}$, and evaluating the complex quantity ${\underline{Z}}_{m}$, which has the dimensions of impedance (trans-impedance), defined as follows:

(1) $${\underline{Z}}_{m}=\frac{{\underline{V}}_{CD}}{{\underline{I}}_{ref}}$$

where

(2) $${\underline{V}}_{CD}=\frac{{\underline{V}}_{AB}}{{\underline{Z}}_{1}+{\underline{Z}}_{2}}*{\underline{Z}}_{2}-\frac{{\underline{V}}_{AB}}{{\underline{Z}}_{T}+{\underline{Z}}_{R}+R_{ref}}*\left({\underline{Z}}_{R}+R_{ref}\right)$$

and

(3) $${\underline{I}}_{ref}=\frac{{\underline{V}}_{AB}}{{\underline{Z}}_{T}+{\underline{Z}}_{R}+R_{ref}}$$

By substituting (2) and (3) into (1), we obtain

(4) $${\underline{Z}}_{m}=\frac{{\underline{Z}}_{2}*{\underline{Z}}_{T}-{\underline{Z}}_{1}*{\underline{Z}}_{R}-{\underline{Z}}_{1}*R_{ref}}{{\underline{Z}}_{1}+{\underline{Z}}_{2}}$$

Consider

(5) $${\underline{Z}}_{T}={\underline{Z}}_{T0}+{\underline{\Delta Z}}_{TA}$$

where ${\underline{Z}}_{T0}$ denotes the intercostal impedance without breath and movement artifacts, and ${\underline{\Delta Z}}_{TA}$ denotes the intercostal impedance variation due to breath and movement artifacts.

Additionally, consider

(6) $${\underline{Z}}_{R}={\underline{Z}}_{R0}+{\underline{\Delta Z}}_{RR}+{\underline{\Delta Z}}_{RA}$$

where ${\underline{Z}}_{R0}$ denotes the esophageal tract impedance without reflux and without breath and movement artifacts, ${\underline{\Delta Z}}_{RR}$ denotes the esophageal tract impedance variation due to reflux, and ${\underline{\Delta Z}}_{RA}$ denotes the esophageal tract impedance variation due to artifacts from breath and movement.

We substitute (5) and (6) into Equation (4):

(7) $${\underline{Z}}_{m}=\frac{{\underline{Z}}_{2}*\left({\underline{Z}}_{T0}+{\underline{\Delta Z}}_{TA}\right)-{\underline{Z}}_{1}*\left({\underline{Z}}_{R0}+{\underline{\Delta Z}}_{RR}+{\underline{\Delta Z}}_{RA}\right)-{\underline{Z}}_{1}*R_{ref}}{{\underline{Z}}_{1}+{\underline{Z}}_{2}}$$

By varying ${\underline{Z}}_{1}$ and ${\underline{Z}}_{2}$, the bridge is initially balanced without the presence of breath and movement artifacts (${\underline{\Delta Z}}_{TA}=0$; ${\underline{\Delta Z}}_{RA}=0$) or reflux episodes (${\underline{\Delta Z}}_{RR}=0$); therefore, in a balanced condition, we have the following:

(8) $${\underline{Z}}_{1}*\left({\underline{Z}}_{R0}+R_{ref}\right)={\underline{Z}}_{2}*{\underline{Z}}_{T0}$$

Using (8) in (7), we have

(9) $${\underline{Z}}_{m}=\frac{{\underline{Z}}_{2}*{\underline{\Delta Z}}_{TA}-{\underline{Z}}_{1}*{\underline{\Delta Z}}_{RR}-{\underline{Z}}_{1}*{\underline{\Delta Z}}_{RA}}{{\underline{Z}}_{1}+{\underline{Z}}_{2}}$$

Considering that ${\underline{Z}}_{R0}$ and ${\underline{Z}}_{T0}$ have very similar values, the bridge will be balanced with ${\underline{Z}}_{1}≅{\underline{Z}}_{2}$. Starting from this condition, when the patient begins to normally breathe and move (${\underline{\Delta Z}}_{TA}≅0$ and ${\underline{\Delta Z}}_{RA}≅0$), we also have ${\underline{Z}}_{2}*{\underline{\Delta Z}}_{TA}≅{\underline{Z}}_{1}*{\underline{\Delta Z}}_{RA}$. Thus, considering the trans-impedance ${\underline{Z}}_{m}$ as a figure of merit to evaluate the presence of reflux, it is possible to assume that the proposed solution compensates for breathing and movement artifacts.

This condition was experimentally verified by looking both at the variations in the voltages (${\underline{V}}_{CD}$ − ${\underline{V}}_{EF}$) and at the variation in ${\underline{Z}}_{m}$ in the presence and absence of breathing and movements.

To conclude, the ${\underline{Z}}_{m}$ value is affected only by reflux episodes (${\underline{\Delta Z}}_{RR}$), allowing for their detection considering the usual daily activities of the patients.

## 4. The Proposed Measurement Procedure

To carry out the tests, the two pairs of electrodes were first connected to the subject undergoing experimentation, and the bridge was balanced in the absence of breathing and movement by varying ${\underline{Z}}_{1}$ and ${\underline{Z}}_{2}$.

To obtain a preliminary characterization of the proposed measurement method, reflux episodes were emulated by considering a subject swallowing acidic or basic substances. Obviously, in this situation, a reverse path of the reflux fluid is considered.

To carry out the tests, various liquids were chosen to investigate different pH values (both acidic and basic solutions). Table 1 shows the chosen substances with their pH values.

The tests were repeated, for all the considered substances, on different subjects (patients) by applying the following three-step procedure: (i) measurements were carried out in the absence of swallowed substances and saliva for a total time of 40 s (in this phase, the patient can normally move and breathe); (ii) at 40 s, the subject swallowed a sip of the liquid under study while maintaining normal behavior regarding movement and breathing; and (iii) the real and imaginary parts, module, and phase of ${\underline{Z}}_{m}$ were evaluated and recorded.

To better highlight the variations in ${\underline{Z}}_{m}$ due to the passage of the different liquids, the percentage variation with respect to a reference impedance value, ${\underline{Z}}_{m\_ref}$ (considered as the mean value of ${\underline{Z}}_{m}$ measured in step i)), was evaluated. In particular, it was evaluated with regard to the real ($\Delta Z_{m\_Re}{}_{\%}$) and imaginary ($\Delta Z_{m\_Im}{}_{\%}$) parts, the module ($|{\underline{Z}}_{m}{|}_{\%}$), and the phase ($Ph{\left({\underline{Z}}_{m}\right)}_{\%}$) of ${\underline{Z}}_{m}$ (Equations (10) to (13), respectively).

(10) $$\Delta Z_{m\_Re}{}_{\%}=\left(\frac{Re\left({\underline{Z}}_{m}\right)-Re\left({\underline{Z}}_{m\_ref}\right)}{Re\left({\underline{Z}}_{m\_ref}\right)}\right)*100$$

(11) $$\Delta Z_{m\_Im}{}_{\%}=\left(\frac{Im\left({\underline{Z}}_{m}\right)-Im\left({\underline{Z}}_{m\_ref}\right)}{Im\left({\underline{Z}}_{m\_ref}\right)}\right)*100$$

(12) $$|Z_{m}{|}_{\%}=\left(\frac{\left|{\underline{Z}}_{m}\right|-\left|{\underline{Z}}_{m\_ref}\right|}{\left|{\underline{Z}}_{m\_ref}\right|}\right)*100$$

(13) $$Ph{\left({\underline{Z}}_{m}\right)}_{\%}=\left(\frac{Ph\left({\underline{Z}}_{m}\right)-Ph\left({\underline{Z}}_{m\_ref}\right)}{Ph\left({\underline{Z}}_{m\_ref}\right)}\right)*100$$

## 5. Measurement Results

Tests were carried out on eight different subjects who swallowed the various chosen liquids (see Table 1). For the sake of brevity, only those results obtained in tests carried out on a medium-sized adult male subject (height 175 cm, weight 72 kg) are reported. Similar results were obtained for the other subjects.

The following values were selected to balance the bridge in the absence of breath, movement artifacts, and swallowing (reflux emulation): R_1_ = 500 Ω, R_2_ = 470 Ω, C_1_ = 7.5 µF, C_2_ = 6 µF.

**Figure 4:**
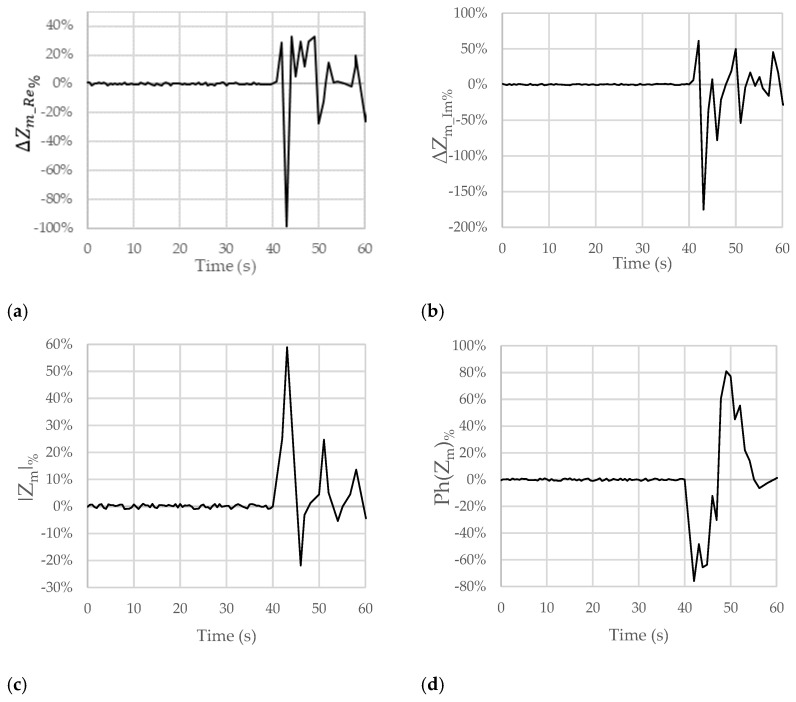
Trends of the chosen figures of merit for $\Delta Z_{m\_Re}{}_{\%}$, $\Delta Z_{m\_Im}{}_{\%}$, $|{\underline{Z}}_{m}{|}_{\%}$, and $Ph{\left({\underline{Z}}_{m}\right)}_{\%}$ when the considered subject swallowed lemon juice (pH = 2.4) at the time of 40 s. The subject moved and breathed normally for the overall duration of the test.

Figure 4 shows the trends of the chosen figures of merit when the considered subject swallowed lemon juice at the time of 40 s. In particular, the real ($\Delta Z_{m\_Re}{}_{\%}$) and imaginary ($\Delta Z_{m\_Im}{}_{\%}$) parts, the module ($|{\underline{Z}}_{m}{|}_{\%}$), and the phase ($Ph{\left({\underline{Z}}_{m}\right)}_{\%}$) of ${\underline{Z}}_{m}$ are, respectively, reported in Figure 4a–d.

Looking at Figure 4a, after the passage of the lemon juice at the time of 40 s, a small and brief increase followed by a significant and clear decrease in $\Delta Z_{m\_Re}{}_{\%}$ of −96% can be observed. A similar behavior can be pointed out for $\Delta Z_{m\_Im}{}_{\%}$, with a variation of −161% (see Figure 4b). In Figure 4c,d, the trends for $|{\underline{Z}}_{m}{|}_{\%}$ and $Ph{\left({\underline{Z}}_{m}\right)}_{\%}$ show percentage variations of about +60% (increase) and −70% (decrease), respectively.

These results allowed us to confirm both the compensation of the artifacts due to breath and movement (since in the time interval from 0 s to 40 s, no significant variations in the considered figures of merit can be observed) and the ability to detect the passage of acid substances (acid reflux).

**Figure 5:**
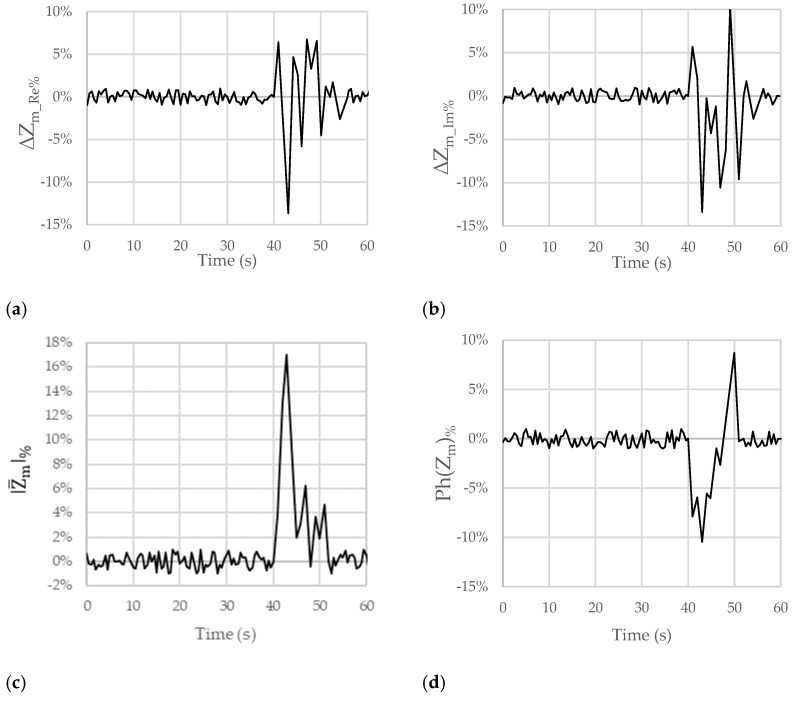
Trends of the chosen figures of merit for $\Delta Z_{m\_Re}{}_{\%}$, $\Delta Z_{m\_Im}{}_{\%}$, $|{\underline{Z}}_{m}{|}_{\%}$, and $Ph{\left({\underline{Z}}_{m}\right)}_{\%}$ when the considered subject swallowed milk (pH = 6.0) at the time of 40 s. The subject moved and breathed normally for the overall duration of the test.

**Figure 6:**
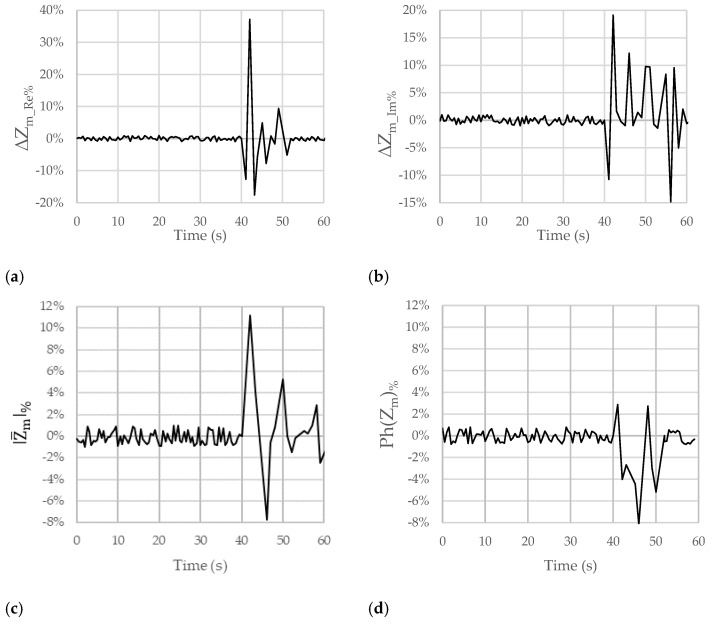
Trends of the chosen figures of merit for $\Delta Z_{m\_Re}{}_{\%}$, $\Delta Z_{m\_Im}{}_{\%}$, $|{\underline{Z}}_{m}{|}_{\%}$, and $Ph{\left({\underline{Z}}_{m}\right)}_{\%}$ when the considered subject swallowed natural water (pH = 7.5) at the time of 40 s. The subject moved and breathed normally for the overall duration of the test.

**Figure 7:**
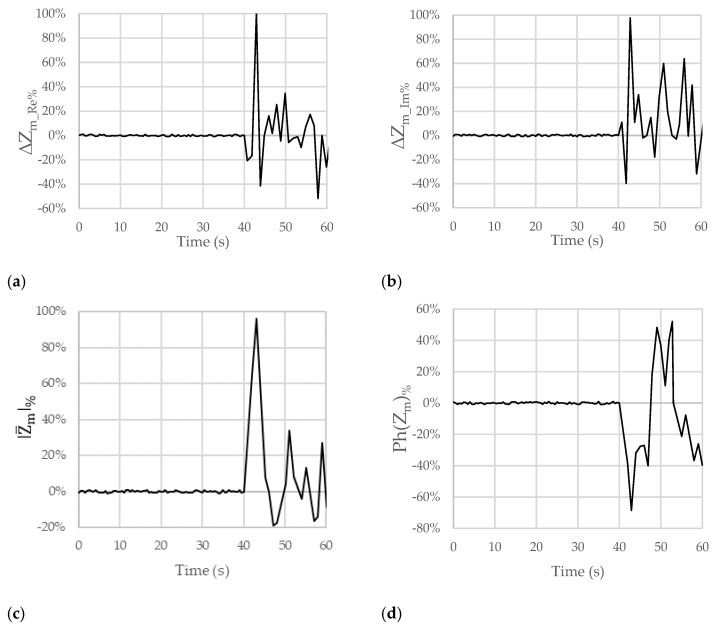
Trends of the chosen figures of merit for $\Delta Z_{m\_Re}{}_{\%}$, $\Delta Z_{m\_Im}{}_{\%}$, $|{\underline{Z}}_{m}{|}_{\%}$, and $Ph{\left({\underline{Z}}_{m}\right)}_{\%}$ when the considered subject swallowed water with sodium bicarbonate (pH = 8.6) at the time of 40 s. The subject moved and breathed normally for the overall duration of the test.

Figure 5, Figure 6 and Figure 7 show the results obtained for the considered figures of merit ($\Delta Z_{m\_Re}{}_{\%}$, $\Delta Z_{m\_Im}{}_{\%}$, $|{\underline{Z}}_{m}{|}_{\%}$, and $Ph{\left({\underline{Z}}_{m}\right)}_{\%}$) in the other analyzed test cases described in Table 1. The behavior is always similar, with changes in terms of both the sign and the amplitude of the percentage variations that can be linked with the pH of the ingested substances.

In particular, Figure 5a–d concerns the data collected in the case of swallowing milk, which has a slightly acidic pH.

The plots in Figure 5a,b show a variation of −14% as regards $\Delta Z_{m\_Re}{}_{\%}$ and a variation of −14% for $\Delta Z_{m\_Im}{}_{\%}$. These variations are smaller than those in the previous case due to the different degree of acidity and, therefore, the higher pH of the substance in this case. In addition, thanks to the smaller scale on the y-axis (from −15% to 10%), the small variations in $\Delta Z_{m\_Re}{}_{\%}$ and $\Delta Z_{m\_Im}{}_{\%}$ due to artifacts (between 0 s and 40 s) can be appreciated. They can be considered negligible with respect to the variations that are due to the emulated reflux episode.

In the plots related to $|{\underline{Z}}_{m}{|}_{\%}$ and $Ph{\left({\underline{Z}}_{m}\right)}_{\%}$ (Figure 5c,d), variations of +16% and −10% can be observed, respectively. Similar considerations can be made for the reduction in the obtained impedance variations and for the negligible variations due to artifacts.

Figure 6a–d shows the results obtained from the measurements made when natural water was ingested. Percentage variations of + 38% for $\Delta Z_{m\_Re}{}_{\%}$, +19% for $\Delta Z_{m\_Im}{}_{\%}$, +11% for $|{\underline{Z}}_{m}{|}_{\%}$, and +3% for $Ph{\left({\underline{Z}}_{m}\right)}_{\%}$ were obtained. Changes in the sign of variation for the figures of merit for $\Delta Z_{m\_Re}{}_{\%}$, $\Delta Z_{m\_Im}{}_{\%}$, and $Ph{\left({\underline{Z}}_{m}\right)}_{\%}$ can be observed. This can be linked to the basicity of the ingested substance.

Figure 7a–d show the results obtained from the measurements carried out when a solution composed of 500 mL of water and 25 g of sodium bicarbonate was ingested. Percentage variations of +100% for $\Delta Z_{m\_Re}{}_{\%}$, +99% for $\Delta Z_{m\_Im}{}_{\%}$, +95% for $|{\underline{Z}}_{m}{|}_{\%}$, and −67% for $Ph{\left({\underline{Z}}_{m}\right)}_{\%}$ were observed.

In this case, the sign of the variation for $\Delta Z_{m\_Re}{}_{\%}$ and $\Delta Z_{m\_Im}{}_{\%}$ is positive, which is linked to the basicity of the ingested substance. Unfortunately, the sign of the variation for $|{\underline{Z}}_{m}{|}_{\%}$ is still positive, while there is a new change in the sign for $\Delta Z_{m\_Im}{}_{\%}$.

Table 2 summarizes the maximum values of the percentage variations in the considered figures of merit in the various cases discussed above.

Looking at Table 2, it can be seen that all the figures of merit were able to portray reflux episodes (with variations due to reflux being bigger than those due to artifacts), but $|{\underline{Z}}_{m}{|}_{\%}$ and $Ph{\left({\underline{Z}}_{m}\right)}_{\%}$ are not able to distinguish between acid and non-acid reflux. Regarding $\Delta Z_{m\_Re}{}_{\%}$ and $\Delta Z_{m\_Im}{}_{\%}$, it is possible to observe a direct correlation between the pH values and the maximum variations in these figures of merit, combined with the ability to detect whether the pH is acid or basic as a result of the sign of the observed variations. In addition, $\Delta Z_{m\_Re}{}_{\%}$ and $\Delta Z_{m\_Im}{}_{\%}$ show greater sensitivity to pH variation.

For these reasons, hereafter, we detail some considerations for only the $\Delta Z_{m\_Re}{}_{\%}$ and $\Delta Z_{m\_Im}{}_{\%}$ figures of merit. Firstly, in Figure 4a,b, Figure 5a,b, Figure 6a,b and Figure 7a,b, there is an initial variation in $\Delta Z_{m\_Re}{}_{\%}$ and $\Delta Z_{m\_Im}{}_{\%}$ at the time of 40 s because swallowing triggers a wave of inhibition of the esophageal smooth muscle that precedes the arrival of the peristaltic contraction, preparing the esophageal body to receive the oncoming bolus [23,24]. Obviously, this happened because we were performing tests involving swallowing; in the case of reflux, this behavior will not occur.

In all the figures, after a variation in greater magnitude, some oscillations follow.

These oscillations are attributable to the destabilization caused by the passage of the substance in the esophageal tract (peristaltic wave). In the case of GERD, a Post-reflux Swallow-induced Peristaltic Wave (PSPW) occurs; therefore, there is a drop in impedance from the proximal to the distal esophagus that can last for up to 30 s. A peristaltic wave is also present when swallowing [25]. In any case, reflux episodes give rise to a variation in the considered trans-impedance ${\underline{Z}}_{m}$ that is bigger than the variation due to the PSPW. In addition, the PSPW always follows a reflux episode; thus, the two variations are time shifted. The magnitude of the oscillations increases as the acidity or basicity of the swallowed substance increases. The oscillations in the figures could also depend on the swallowing of saliva by the patient to mitigate the taste associated with the ingestion of the substance. In the first 40 s, nothing was swallowed, not even saliva. On average, the pH of saliva approaches neutrality, oscillating between 6.5 and 7.4 as a result of the buffering action of the bicarbonates it contains. Positive and negative variations are, however, quite frequent and physiological in most cases. The pH of saliva is, in fact, influenced by the degree of oral hygiene, the type of diet, etc.

Milk and natural water have a pH similar to that of saliva; therefore, the variations in the figures of merit due to saliva ingestion are similar to those due to the ingestion of these substances. This is a limitation of the proposed method since it is difficult to detect reflux episodes with pH near neutral. However, the invasive devices currently used and discussed in Section 2 suffer the same limitation.

## 6. Conclusions

A non-invasive technique for the detection of gastric reflux was proposed here. It is based on the use of two pairs of electrodes externally attached to the trunk of the patient and on the measurement of a particular quantity defined as the trans-impedance (${\underline{Z}}_{m}$). The proposed solution allows for long-term monitoring during normal daily life as a result of its ability to compensate for artifacts related to breathing and movement. It was shown through experiments that the proposed technique is able to establish a correlation between the pH of the reflux and the real and imaginary parts of the complex measured trans-impedance. The other analyzed figures of merit (modulus and phase of the trans-impedance) failed to give satisfactory results due to their lower detection sensitivity and inability to distinguish between acidic and basic reflux.

At low pH values (acidic reflux), negative variations in the order of 100% or higher were associated with the real and imaginary parts of ${\underline{Z}}_{m}$; in the case of high pH (basic reflux), on the other hand, a positive variation with a similar order of magnitude was observed. In intermediate cases of slightly acidic or slightly basic substances, the trends resembled those of strongly acidic or basic substances, but the variations were always smaller when approaching neutral pH values.

The goodness of the results demonstrated the validity of the proposed method.

## 7. Actual Limitation and Future Developments

This study focused on presenting a new measurement method and validating it by means of a preliminary experimental campaign emulating reflux episodes involving subjects swallowing acidic or basic substances.

This approach allowed us to easily and quickly make a preliminary validation of the proposed solution, but a detailed experimental campaign involving different subjects suffering gastroesophageal reflux is still needed. In particular, a comparison with the results obtained using a gold-standard instrument is still missing. The obtained results showed that the proposed solution seems to be unable to solve the problem related to the detection of neutral pH reflux episodes that can be confused with the swallowing of saliva or with esophageal muscle activity. In any case, this limitation is shared with the invasive techniques currently in use.

Future work will focus on carrying out further tests in real conditions with patients affected by reflux disorder and comparing the results with those obtained via the parallel use of gold-standard instrumentation such as impedance-pH testing and/or other invasive techniques in an ambulatory environment. Finally, after the realization of a portable version of the proposed monitoring system, this will be used to monitor reflux patients in conjunction with other systems, allowing for a full comparison of the results in a real continuous monitoring condition.

## Figures and Tables

**Table 1:** pH values of the substances adopted in the experimental tests.

Substance	pH
Lemon juice	2.4
Skimmed milk	6.0
Natural water	7.5
Water (500 mL) with sodium bicarbonate (25 g)	8.6

**Table 2:** Maximum variations in the considered figures of merit for substances with different pH.

Substance	pH	$\Delta Z_{m\_Re}{}_{\%}$	$\Delta Z_{m\_Im}{}_{\%}$	$|{\underline{Z}}_{m}{|}_{\%}$	$Ph{\left({\underline{Z}}_{m}\right)}_{\%}$
Lemon juice	2.4	−96%	−161%	+60%	−70%
Skimmed milk	6.0	−14%	−14%	+16%	−10%
Natural water	7.5	+38%	+19%	+11%	+3%
Water (500 mL) with sodium bicarbonate (25 g)	8.6	+100%	+99%	+95%	−67%

## Data Availability

All data are contained within the article.
